# Supplementary material for: Effects of Five Substances with Different Modes of Action on Cathepsin H, C and L Activities in Zebrafish Embryos
Source: Int J Environ Res Public Health. 2019 Oct 17;16(20):3956. doi: 10.3390/ijerph16203956 (PMC6843663; doi:10.3390/ijerph16203956)
Supplement: Supplementary file 1 [file ijerph-16-03956-s001.pdf]

**SUPPLEMENTARY DATA** (Küster et al. Cathepsins in zebrafish embryos)

## 1. ESI-MS/MS based identification of upregulated protein bands from 1D SDS-Experiment (Figure 2B)

### 1.1 Method

The two protein bands (fragment a and fragment b from 0.2 mM leupeptin exposure [Figure 2B]) of interest were excised from stained gels, destained, and prepared for nano-HPLC/nano-ESI-MS/MS analysis by reduction, alkylation, tryptic digestion, peptide extraction, and drying. Peptides were reconstituted in 0.1% formic acid. The measurements were performed using a nano-HPLC system (nanoAcquity, Waters) online coupled to a linear ion trap/orbitrap hybrid mass spectrometer (LTQ orbitrap XL ETD, Thermo Fisher Scientific). Briefly, samples were injected by the autosampler and concentrated on a trapping column (nanoAcquity UPLC column, C18, 180 µm x 20 mm, 5 µm, Waters) with 0.1% formic acid at a flow rate of 15 µL/min. After 4 min, the peptides were eluted onto the separation column (nanoAcquity UPLC column, C18, 75 µm x 150 mm, 1.7 µm, Waters). Chromatography was performed using 0.1% formic acid in solvents A (100% water) and B (100% ACN) with peptides eluting over 30 min with 8–40% solvent B gradient. Continuous scanning of eluted peptide ions was carried out between *m/z* 350–2000*m/z*, automatically switching to MS/MS CID mode on ions exceeding an arbitrary intensity of 2000 counts. Raw MS/MS spectra were converted to Mascot generic files (mgf-files) and database searches by MASCOT search engine were performed using the ProteomDiscoverer 1.0 software (Thermo Fisher Scientific). Searches were conducted on NCBI database against all *Danio rerio* sequences, tolerating up to one tryptic missed cleavage, a mass tolerance of 10 ppm for precursor ions and 0.5 Da for MS/MS product ions allowing for methionine oxidation (dynamic modification), and cysteine carbamidomethylation (static modification).

| ***Band Name*** | ***Protein Name*** | ***Species*** | ***MASCOT Score*** | ***Database*** | ***NCBI ID / GenBank ID*** | ***MW (kDa) obs/cal*** |
|---|---|---|---|---|---|---|
| 0.2 mM Leupeptin fragment a | Vitellogenin 1 fragment a | *Danio rerio* | 5317 | NCBI | gi\|94733730 / CAK03614 | 100/149.8 |
| 0.2 mM Leupeptin fragment b | Vitellogenin1 fragment b | *Danio rerio* | 4399 | NCBI | gi\|94733730 / CAK03614 | 66/149.8 |

### 1.2 Results

### Mascot Search Results

## Vitellogenin 1 fragment a:

Match to: **gi|94733730** Score: **5317**
**vitellogenin 1 [Danio rerio]**

Nominal mass ($M_r$): **149825**; Calculated pI value: **8.74**
NCBI BLAST search of gi|94733730 against nr
Unformatted sequence string for pasting into other applications

Taxonomy: Danio rerio

Fixed modifications: Carbamidomethyl (C)
Variable modifications: Oxidation (M)
Cleavage by Trypsin: cuts C-term side of KR unless next residue is P
Sequence Coverage: **46%**

Matched peptides shown in **Bold Red**

**1** MRAVVLALTV ALVACQQFNL VPEFAHDK**TY VYKYEALLLG GLPQEGLAR**A
**51** GIKVSSK**VLI SATTENTYLM KLMDPLLYEY AGTWPKDPFV PATKLTSALA**
**101** **AQLQIPIKFE YANGVVGKVF APAGVSPTVM NLHRGILNIL QLNLKK**TQNI
**151** YEMQEAGAQG VCR**THYVINE DPKANHIIVT K**SKDLSHCQE RIMK**DVGLAY**
**201** **TER**CAECTER VKSLIETATY NYIMKPADNG ALIAEATVEE VYQFSPFNEI
**251** HGAAMMEAK**Q TLAFVEIEKT PVVPIKADYM PRGSLQYEFA TEILQTPIQL**
**301** **MKISDAPAQI VEVLKHLVSN NKDMVHDDAP FKFVQLVQLL R**VASLEK**IEA**
**351** **IWSQFKDKPV YRRWLLDALP AVGTPVIIK**F IKEK**FLAGEF TTPEFIQTLV**
**401** **IALQMVTADP ETIKMTASLA THEKFATIPA LREVVMLGYG SLIAK**YCVAV
**451** PTCPAELLRP IHEIATEAIS K**NDIPEITLA LKVMGNAGHP SSLKPIMKLL**
**501** **PGLRTAANAL PIRVQVDAIL ALR**NIAKKEP K**LVQPVALQL VLDRALHPEV**
**551** **R**MVACIVLFE AEPSVALVSS LAGALR**IEPN MHVASFAYSH IK**SLTR**ITAP**
**601** **DMASVAGAAN VAIKLMSR**KL DRLNYRYSR**A FQMDYYYTPL MIGAAGSAYM**
**651** **INDAATILPR** AVVAKAR**AYL AGAAADVIEF GVRTGGIHEA LLKSPAADES**
**701** **ADRITK**IKRT LR**ALTNWKAL PTDKPLASAY LKVFGQEVAY VNFDKTIIEE**
**751** **AIPMATGPKP R**ALLKEALK**A LQEGVAFQYA KPLLAAEVR**R ILPTAVGVPM
**801** EFSWYTAAVA AASVNVQATI TPALPEK**LES MTYEQLKK**TD VQFQAEARPS
**851** VALQTFAVMG VNTAFIQAAV MARGKIRTIA PGKVAAR**ADI LKGNYKVEAL**
**901** **PVELPEHIAS ASFETYAVVR NIEDHSAER**S VPLVPELSLQ NSQASYAGDL
**951** SSEMSSVASV RAPAPFDRTL CYAVPYIEIK GCVEVHSHNA AFIRNSTLFY
**1001** IIGHHSVRAA VARAEGPAVE RLEFEVQVGP RAAERLVKQI NIIDDDTPEG
**1051** QAFLLKLREI LDTEAKNAPV SSESSSSRNS RSSSSRSTST STSSSSSSSS
**1101** SSSSSSSSSM SSSRMSKTAT IIEPFRKFHK DRYLAHHSAT KDTSSGSAAA
**1151** SFEQMQKQNR **FLGNDIPPVF AIIAR**AVRAD QKLLGYQLAA YFDKPTARVQ
**1201** LIVSSIAEND NMKICADGAL LSKHKVTGKF SWGAECKQYA VFAKAEAGVL
**1251** GEFPAARLEV EWER**LPIIVT TYAK**KLGKHI LTAAYDTGFR FERATNSEKE
**1301** IELTAALPSQ RSLNIIARIP EITMSKRDIY LPVAVPINPD GTFSIETYED
**1351** FLAWIQKYIK EE

---

## Vitellogenin 1 fragment b:

Match to: **gi|94733730** Score: **4399**
**vitellogenin 1 [Danio rerio]**

Nominal mass ($M_r$): **149825**; Calculated pI value: **8.74**
NCBI BLAST search of gi|94733730 against nr
Unformatted sequence string for pasting into other applications

Taxonomy: Danio rerio

Fixed modifications: Carbamidomethyl (C)
Variable modifications: Oxidation (M)
Cleavage by Trypsin: cuts C-term side of KR unless next residue is P
Sequence Coverage: **46%**

Matched peptides shown in **Bold Red**

**1** MRAVVLALTV ALVACQQFNL VPEFAHDK**TY VYKYEALLLG GLPQEGLAR**A
**51** GIKVSSK**VLI SATTENTYLM KLMDPLLYEY AGTWPKDPFV PATKLTSALA**
**101** **AQLQIPIKFE YANGVVGKVF APAGVSPTVM NLHRGILNIL QLNLKK**TQNI
**151** YEMQEAGAQG VCR**THYVINE DPKANHIIVT K**SKDLSHCQE RIMK**DVGLAY**
**201** **TER**CAECTER VKSLIETATY NYIMKPADNG ALIAEATVEE VYQFSPFNEI
**251** HGAAMMEAK**Q TLAFVEIEKT PVVPIKADYM PRGSLQYEFA TEILQTPIQL**
**301** **MKISDAPAQI VEVLKHLVSN NKDMVHDDAP FKFVQLVQLL RVASLEKIEA**
**351** **IWSQFKDKPV YRRWLLDALP AVGTPVIIK**F IKEK**FLAGEF TTPEFIQTLV**
**401** **IALQMVTADP ETIKMTASLA THEKFATIPA LREVVMLGYG SLIAK**YCVAV
**451** PTCPAELLRP IHEIATEAIS K**NDIPEITLA LKVMGNAGHP SSLKPIMKLL**
**501** **PGLRTAANAL PIRVQVDAIL ALR**NIAKKEP K**LVQPVALQL VLDRALHPEV**
**551** **R**MVACIVLFE AEPSVALVSS LAGALR**IEPN MHVASFAYSH IKSLTRITAP**
**601** **DMASVAGAAN VAIKLMSR**KL DRLNYRYSR**A FQMDYYYTPL MIGAAGSAYM**
**651** **INDAATILPR** AVVAKAR**AYL AGAAADVIEF GVRTGGIHEA LLKSPAADES**
**701** **ADR**ITKIKRT LRALTNWK**AL PTDKPLASAY LKVFGQEVAY VNFDKTIIEE**
**751** **AIPMATGPKP R**ALLKEALK**A LQEGVAFQYA KPLLAAEVR**R ILPTAVGVPM
**801** EFSWYTAAVA AASVNVQATI TPALPEK**LES MTYEQLKK**TD VQFQAEARPS
**851** VALQTFAVMG VNTAFIQAAV MARGKIRTIA PGKVAARADI LKGNYK**VEAL**
**901** **PVELPEHIAS ASFETYAVVR NIEDHSAER**S VPLVPELSLQ NSQASYAGDL
**951** SSEMSSVASV RAPAPFDRTL CYAVPYIEIK GCVEVHSHNA AFIRNSTLFY
**1001** IIGHHSVRAA VARAEGPAVE RLEFEVQVGP RAAERLVKQI NIIDDDTPEG
**1051** QAFLLKLREI LDTEAKNAPV SSESSSSRNS RSSSSRSTST STSSSSSSSS
**1101** SSSSSSSSSM SSSRMSKTAT IIEPFRKFHK DRYLAHHSAT KDTSSGSAAA
**1151** SFEQMQKQNR FLGNDIPPVF AIIARAVRAD QKLLGYQLAA YFDKPTARVQ
**1201** LIVSSIAEND NMKICADGAL LSKHKVTGKF SWGAECKQYA VFAKAEAGVL
**1251** GEFPAAR**LEV EWERLPIIVT TYAK**KLGKHI LTAAYDTGFR FERATNSEKE
**1301** IELTAALPSQ RSLNIIAR**IP EITMSK**RDIY LPVAVPINPD GTFSIETYED
**1351** FLAWIQKYIK EE
